# Retraction Note to: miR-484 suppresses proliferation and epithelial–mesenchymal transition by targeting ZEB1 and SMAD2 in cervical cancer cells

**DOI:** 10.1186/s12935-021-01958-0

**Published:** 2021-05-10

**Authors:** Yang Hu, Hong Xie, Yankun Liu, Weiying Liu, Min Liu, Hua Tang

**Affiliations:** Tianjin Life Science Research Center and Department of Pathogen Biology, School of Basic Medical Sciences, Tianjin Medical University, 22 Qi‑Xiang‑Tai Road, Tianjin, 300070 China

## Retraction to: Cancer Cell Int (2017) 17:36 https://doi.org/10.1186/s12935-017-0407-9

The Editors-in-Chief have retracted this article [1]. Concerns were raised regarding a number of figures, specifically:

- Figure 3a: the first and third panel for C33A appear to partially overlap.
- Figure 3b: panel 2 for C33A appears to partially overlap with panel 2 for C33A in Figure 7b.
- Figure 7a: panel 1 for HeLa appears to overlap with panel 3 for HeLa in Figure 7b.
- Figure 7a: panel 3 for C33A appears to partially overlap with panels 4 and 4 for C33A in Figure 7b: panel 6 for HeLa appears to partially overlap with panel 5 for C33A.

The Editors-in-Chief therefore no longer have confidence in the reliability of the data reported in the article.

All authors agree to this retraction.
